# Screening Cardiovascular Risk in Patients with High Depression Scores

**DOI:** 10.12669/pjms.343.14560

**Published:** 2018

**Authors:** Burkay Yakar, Yusuf Haydar Ertekin

**Affiliations:** 1 Dr. Burkay Yakar, Department of Family Medicine, Firat University School of Medicine, Elazig, Turkey; 2 Dr. Yusuf Haydar Ertekin, Department of Family Medicine, Canakkale Onsekiz Mart University School of Medicine, Canakkale, Turkey

**Keywords:** Beck Depression Inventory, Cardiovascular Risk, Framingham Risk Score, Obesity, Primary Healthcare Services

## Abstract

**Objective:**

Protection against cardiovascular diseases is provided by the dynamics of risk screening and counseling of primary health care services. Depression is known to pose a risk for cardiovascular diseases. The aim of our study was to determine which specific features of well-known depressed people who had not yet experienced a cardiovascular event were associated with cardiovascular risk.

**Methods:**

This study was conducted in patients at the Corum Gulabibey Family Health Center between June 2016 and June 2017. Patients without a known cardiovascular disease were subjected to Beck Depression Inventory (BDI) and Framingham risk scale. Framingham risk scores were compared by dividing the participants into two groups according to having Beck depression scores of equal/above 10 points (high BDI = HBDI) and below 10 points (low BDI = LBDI).

**Results:**

Age, LDL, total cholesterol, triglyceride, and blood pressure were all correlated with risk scores. In contrast, HDL and body mass index were only correlated with the risk scores in HBDI participants. From the HBDI patients, those who were obese had higher risk scores than those without obesity.

**Conclusion:**

Obesity is a high cardiovascular risk predictor that can be screened at one site in depression. While the body mass index of depressed individuals was correlated with the cardiovascular risk, this index being above 30 was indicative of high cardiovascular risk.

## INTRODUCTION

Coronary heart disease (CHD) is an important cause of mortality and morbidity worldwide.^1^ According to American and European data, cardiovascular diseases are the leading causes of death and disability.^1^-^3^ According to follow-up results of the TEKHARF study conducted in Turkey, covering the years 1990-2008, Turkey is prominent among the European countries in deaths due to coronary heart disease with a ratio of 7.64 for men and 3.84 for women.^4^ Hence, the increasing frequency of cardiovascular diseases has made it imperative to identify and reduce the risks in the struggle against this disease.^1^,^5^

Although many risk factors are mentioned for cardiovascular diseases, hypertension, diabetes, obesity, hyperlipidemia, smoking, and sedentary and stressful lifestyles are the most important and modifiable risk factors.^6^ Depression, one of the most disabling diseases worldwide^7^,^8^, has been associated with cardiovascular conditions for the first time^9^ in 1993, and depression has been shown to increase mortality in cardiovascular disease.^10^,^11^ Some meta-analyses have shown that major depression increases the risk of mortality and morbidity in cardiovascular disease.^10^,^12^ Studies conducted in Western countries have demonstrated that workload and stress are related to cardiovascular disease.^13^ At a 10-year follow-up, when the meta-analysis of 21 prospective studies was evaluated, the risk of coronary disease was 1.81-fold higher in depressive patients.^10^ In another similar study, depressive mood was found to increase the risk of CHD by 1.5 times and major depression by 2.7 times.^14^

Patient density in the outpatient clinic service, as well as having a high number of patients with modifiable risk factors such as obesity and depressive mood status, makes it difficult to inquire about CHD risk factors in daily practice. In this study, we investigated how to screen CHD risk at a glance utilizing the relationship between depression scores and CHD risks in outpatient conditions.

## METHODS

**Fig.1:**
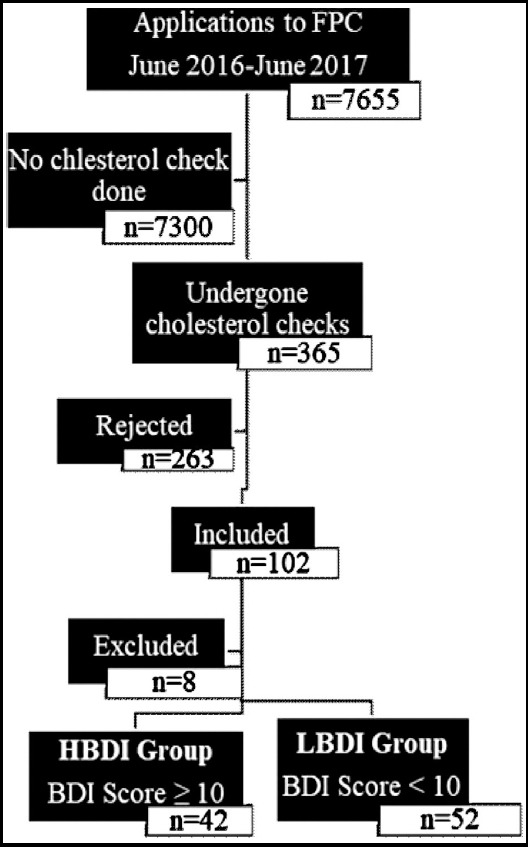
Patient flow diagram.

In this cross-sectional study, patients were approached in the Corum Gulabibey Family Health Center between June 2016 and June 2017 (Fig.1). Patients who had undergone total cholesterol (TC), HDL cholesterol (HDL-C), LDL cholesterol (LDL-C) and triglyceride (TG) tests for some reason were asked to participate on a voluntary basis. Of these patients (n = 365), those with a cardiovascular disease (n = 263) were excluded. The patients were evaluated in a quiet room. Patients’ blood pressure (BP), height, and weight were measured, and body mass index (BMI) was calculated. A family history of CHD, hypertension (HT), diabetes (DM), and smoking status were questioned.

The Beck Depression Inventory (BDI) and Framingham CHD risk scoring were applied to all patients. BDI scores of 10 and above were grouped as high BDI (HBDI), and those below ten were categorized as low BDI (LBDI). Additionally, patients were divided into four groups according to Framingham CHD risk scoring as “very low”, “low”, “moderate”, and “high”. Statistical analyzes were done according to these groupings.

### Beck Depression Inventory (BDI)

BDI is a 20-item scale, scoring between 0-3 for each item, where 10-points or over are indicative of depression, and a score of less than ten is considered as normal.^15^

### Framingham Risk Scoring

Framingham Risk Scoring is a scoring and risk-ratio calculation method that predicts an absolute risk of CHD event for ten years according to age, gender, TC, TG, LDL-C, HDL-C, systolic BP, diastolic BP, and diabetes. Framingham scores are categorized as below 10% for very low risk, 10-15% for low risk, between 15-20% for moderate risk, and above 20% for high risk.^16^

### Ethics and Consent

The study was approved by the Clinical Investigations Ethical Committee of the Canakkale Onsekiz Mart University in the meeting held on March 16^th^ 2016 with reference ID 05-07. Additionally, permission was taken from the Corum Public Health Directorate on 21/04/2016 to conduct the study at the relevant family health center.

**Fig.2:**
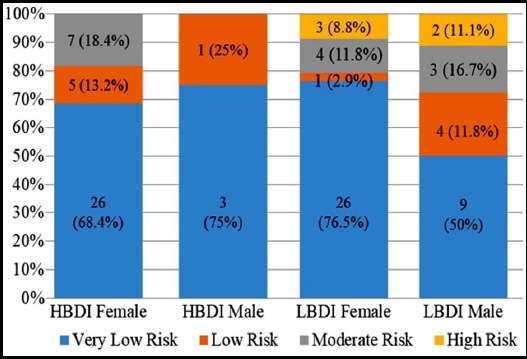
10-year absolute risk stratification of CHD event by groups. HBDI: Beck Depression Inventory Scores ≥10; LBDI: Beck Depression Inventory Scores <10.

### Statistical Analyses

The Statistical Package for Social Sciences (SPSS, Inc., Chicago, IL, USA) for Windows 19 was used for statistical analysis. Descriptive statistics in addition to the Mann Whitney-U test were employed when comparing means, and correlation analysis was used to check for relationships between continuous variables. Categorical variables were compared with the Chi-Square or the Fisher’s exact tests. The results were presented as mean ± SD in 95% confidence intervals. P values <0.05 were considered statistically significant.

## RESULTS

In total, data for 94 patients were analyzed. 22 patients (23.4%) were males, 72 patients (76.6%) were females; mean age was 47.8 years (31 - 63). Of the patients, 42 (44.7%) were in the HBDI group while the remaining 52 (45.3%) were in the LBDI group. The number of females was higher both in HBDI and LBDI groups (Table-I). However, the ratio of women in the HBDI group was significantly higher (Table-I).

**Table-I:** Baseline participant characteristics.

		HBDI	LBDI	p
Age	mean ± SD	46.1 ± 8.7	49.1 ± 8.7	0.099
Gender	Male, n (%)	4 (9.5)	18 (34.6)	0.006*
	Female, n (%)	38 (90.5)	34 (65.4)	
Family history of CVD	n (%)	15 (35.7)	16 (30.8)	0.663
Smoking	n (%)	12 (28.6)	6 (11.5)	0.063
Hypertension	n (%)	10 (23.8)	10 (19.2)	0.620
Diabetes Mellitus	n (%)	11 (26.2)	14 (26.9)	1.000
HDL-C	mean ± SD	46.4 ± 9.3	46.0 ± 13.9	0.861
LDL-C	mean ± SD	132.8 ± 32.7	135.7 ± 34.8	0.681
Total Cholesterol	mean ± SD	208.2 ± 40.2	211.6 ± 40.2	0.683
Triglyceride	mean ± SD	145.5 ± 73.2	146.4 ± 75.1	0.954
Systolic pressure	mean ± SD	128.5 ± 26.0	121.9 ± 18.7	0.170
Diastolic pressure	mean ± SD	79.0 ± 14.2	75.2 ± 12.1	0.170
BMI				
Normal weight (<25 kg/m2)	n (%)	5 (11.9)	4 (7.7)	0.507
Overweight (25 - <30 kg/m2)	n (%)	13 (31.0)	18 (34.6)	0.826
Obese (30 kg/m2 or higher)	n (%)	24 (57.1)	30 (57.7)	1.000
Framingham score	mean ± SD	6.9 ± 5.9	8.7 ± 7.7	0.212
BDI score	mean ± SD	14.7 ± 4.3	4.9 ± 2.4	<0.001

CVD: Cerebrovascular Disease.

Although there were patients with high risk for CHD in the LBDI group, there was no significant difference in the mean risk scores between the two groups (z = -1.100, p = 0.271). In the HBDI group, patients with obesity, DM or HT had a significantly higher risk for CHD, whereas in the LBDI group those with HT, DM, or male sex had a significant risk for CHD (Table-II).

**Table-II:** Comparison of Framingham risk scores in CHD risk-related factors by groups.

Factors	HBDI				LBDI			

	n	mean rank	Z	p	n	mean rank	Z	p
Gender Male	4	24.6	-0.540	0.589	18	33.3	-2.365	0.018
Gender Female	38	21.2			34	22.9		
Normal Weight	5	14.3	-1.409	0.159	4	35.1	-1.189	0.234
Overweight, (BMI=25-29)	13	17.1	-1.563	0.118	18	24.5	-0.685	0.493
Obesity, (BMI>30)	24	25.4	-2.383	0.017	30	26.5	-0.019	0.985
Family history of CHD	15	20.1	-0.542	0.588	16	26.2	-0.109	0.913
Smoking	12	19.7	-0.603	0546	6	28.8	-0.402	0.687
Hypertension	10	30.7	-2.724	0.005	10	40.7	-3.309	0.001
Diabetes Mellitus	11	33.2	-3.705	<0.001	14	35.6	-2.640	0.008

Z: Mann-Whitney U Test value

No significant correlation was found between mean BDI scores [min.-max.] of all patients (9.5 ± 6.8 [0-23]) and mean Framingham scores [min.-max.] (7.9 ± 6.9 [1-31]) (r=-0.063, p=0.546). However, there was a positive correlation between age, LDL, TC, TG, BP, and CHD (Table-II).

When we compared the two groups, there was a positive correlation between BMI and CHD risk in HBDI patients and a negative correlation between HDL and CHD risk in LBDI patients (Table-III).

**Table-III:** Comparison of correlations of CHD risk-related factors with Framingham risk scores by groups.

Factors	HBDI		LBDI	

	r	p	r	p
BDI score	0.189	0.231	-0.267	0.056
Age	0.808	<0.001	0.732	<0.001
HDL	-0.096	0.231	-0.453	0.001
LDL	0.528	<0.001	0.508	<0.001
Total Cholesterol	0.538	<0.001	0.443	0.001
Triglyceride	0.407	0.008	0.490	<0.001
Systolic Pressure	0.475	0.001	0.606	<0.001
Diastolic Pressure	0.372	0.015	0.385	0.005
BMI	0.440	0.004	0.043	0.760

r: Spearman correlation coefficient

## DISCUSSION

Our study revealed a positive correlation between CHD risk scores in both groups with age, LDL, TC, TG, and blood pressure. Obesity, hyperlipidemia, smoking, HT, family history, and DM are known risk factors for CHD.^17^,^18^ Cholesterol, HT, cigarettes, DM, triglycerides, HDL, age, and family history are found as independent risk factors in many studies comparing risk factors.^19^-^24^ Among them, age is positively correlated with depressive symptoms as well.^25^ Our findings of correlations with CHD risk scores and LDL, TC, TG, age, and blood pressure are consistent with the literature. Positive association of these risk factors emerges as an expected outcome, and we believe that correlations with CHD risk scores in both groups demonstrate the significance of our findings.

One remarkable finding in our study is that the BMI of the HBDI group is positively correlated with CHD risk and that those who are particularly obese from HBDIs are at risk for CHD. In a study conducted in China, major depression was found to be a factor related with CHD, but BMI in the depressive patient group was lower than that in the non-depressive patient group^26^, which is contradicting our findings. In this study, HBDI patients were found to be more sedentary and more likely to smoke than the other group, and the increased risk of CHD was associated with these factors. There was no difference between the two groups concerning smoking cessation associated with CHD risk in our research. In a study exploring nine systematic reviews; smoking, low physical activity, and poor lifestyle were reported to be associated with CHD risk in depressing, but the relationship between obesity and BMI was not addressed.^27^ Everson-Rose et al. reported that depressive symptoms may increase cardiovascular disease (CVD) risk by increasing visceral adipocytes in middle-aged women.^28^ However, waist circumference is not a good indicator for visceral adiposity and is highly correlated with BMI (r=0.93). Our findings support that associated with visceral adiposity, BMI may increase cardiovascular risk in depressive patients.

In our study, there was a significant correlation between all cholesterol values in the two groups and the risk of CHD in accordance with the literature^16^,^29^,^30^, whereas in HBDI group there was no inverse correlation between HDL and CVD, which seems to conflict with the literature.^31^,^32^ This contradiction can be explained by the fact that our study has a limitation due to the low number of patients have been included. Keeping in mind this contradiction, further studies with large sample size are needed to explain this situation.

According to our results, the association of HT and DM with CHD risk in both groups was significant. Many studies reveal that HT and DM are independent risk factors for CHD.^33^,^34^ However, according to a meta-analysis, it is reported that depression is strongly related to CVD and DM, and not related to HT.^35^ In our study, co-morbidities with DM in the majority of participants with HT may have produced this result.

## CONCLUSION

In individuals with high BDI scores, there is an apparent relationship between BMI of 30 and above and CVD risk, and a positive correlation between BMI and CVD risk. Further studies need to be conducted in larger patient populations to confirm our findings and to determine which factors other than obesity are important in depression.

### Author`s Contribution

**BY, YHE** conceived, designed, manuscript writing, and final approval of manuscript.

**BY** did data collection.

**YHE** did statistical analysis, editing of manuscript.
